# Cats, Cancer and Comparative Oncology

**DOI:** 10.3390/vetsci2030111

**Published:** 2015-06-30

**Authors:** Claire M. Cannon

**Affiliations:** University of Tennessee College of Veterinary Medicine, 2407 River Drive, Knoxville, TN 37996, USA; E-Mail: clairemcannon@gmail.com

**Keywords:** comparative oncology, feline cancer, animal models

## Abstract

Naturally occurring tumors in dogs are well-established models for several human cancers. Domestic cats share many of the benefits of dogs as a model (spontaneous cancers developing in an immunocompetent animal sharing the same environment as humans, shorter lifespan allowing more rapid trial completion and data collection, lack of standard of care for many cancers allowing evaluation of therapies in treatment-naïve populations), but have not been utilized to the same degree in the One Medicine approach to cancer. There are both challenges and opportunities in feline compared to canine models. This review will discuss three specific tumor types where cats may offer insights into human cancers. Feline oral squamous cell carcinoma is common, shares both clinical and molecular features with human head and neck cancer and is an attractive model for evaluating new therapies. Feline mammary tumors are usually malignant and aggressive, with the ‘triple-negative’ phenotype being more common than in humans, offering an enriched population in which to examine potential targets and treatments. Finally, although there is not an exact corollary in humans, feline injection site sarcoma may be a model for inflammation-driven tumorigenesis, offering opportunities for studying variations in individual susceptibility as well as preventative and therapeutic strategies.

## 1. Introduction

‘Cats are not small dogs’ is an oft-quoted phrase in veterinary medicine. Nowhere is this more true than in comparative oncology, and it is both the advantage and disadvantage of the cat as a model.

Although domestic cats have been used as models for various non-neoplastic diseases and are of particular value for investigation of inherited ophthalmic diseases and type 2 diabetes [1,2], to date the dog has been the focus in comparative oncology [3,4]. This may be due, in part, to the fact that the complete feline genome has only very recently become available [5], whereas the canine genome has been available since 2005 [6]. The resources are now available to begin to fully investigate naturally-occurring cancers in cats as models for human diseases beyond the clinical and histological similarities. Greater coverage of the feline genome and development of a feline microarray chip similar to those developed for humans and dogs, allowing genome-wide assessments, would assist in fully investigating cats as cancer models.

Cancer is common in domestic cats, though likely somewhat less common than in dogs [7,8]. Like dogs, cats with cancer have clear benefits over laboratory models of human cancers (*i.e.*, induced tumors or xenografted human cell lines in rodents). Cats and dogs experience the same environmental risk factors as humans and are immunocompetent, more accurately reflecting the complex interplay of genetics and environmental risk factors as well as the role of the immune system and tumor microenvironment. There is greater homology between dogs and humans for key cancer-related genes than there is between rodents and humans [9], and there is also significant homology between cats and humans for specific genes [10,11,12]. Comparative genomics in feline cancers are reviewed in detail elsewhere in this issue [13]. The shorter lifespan and more rapid progression of cancers in animals allows more rapid trial completion and data collection, potentially identifying therapies that are more likely to succeed in human trials. There is a lack of standard of care for many cancers in animals and new therapies are more likely to be evaluated in treatment-naïve patients, which may give better determination of toxicity and efficacy. The cat may be superior to the dog as a model for some specific tumors, e.g., oral squamous cell carcinoma (SCC) and aggressive mammary tumors, due to the increased frequency in this species. Sarcoma formation secondary to inflammation (at injection sites or following trauma), while rarely reported in dogs, is well-recognized in cats, and may offer insights into inflammation-driven tumorigenesis in general. Published feline cell lines for these tumor types are included in Table 1.

When considering potential benefits of the cat as a model, there are some species-specific challenges to be considered. Although 2012 data suggest that there are more cats than dogs owned as pets in the United States, they are less likely to be seen by veterinarians, either at primary care or referral hospitals [14,15,16,17,18]. This effect is more pronounced in cats greater than 9 years of age [17], meaning that cats at highest risk of cancer are least likely to be seen for routine veterinary care. Cat owners are more likely to perceive their pet being stressed by veterinary visits than dog owners [17]. In the specific situation of clinical trials, a recent survey found that most cat owners were unsure about whether they would consider enrolling their cat in a hypothetical trial, though 71% of owners who had previously participated in a clinical trial would consider participating again. Important factors in owners’ decisions included the recommendation of their primary veterinarian, the number of visits, and the risk of discomfort to their cat [19]. Although there are barriers to clinical trials in cats, there are opportunities at every level to improve recruitment including education of the public about the importance of routine veterinary care and the existence of clinical trials, of primary care veterinarians about clinical trial opportunities and of both primary care and specialist veterinarians about ‘feline-friendly’ or low-stress handling techniques.

One of the strengths of the dog in comparative oncology is the relatively genetically homogenous breeds (compared to humans) with breed predispositions to different cancers, simplifying identification of genetic signatures related to cancer predisposition (for example, DNA repair deficiency in Golden Retrievers associated with higher risk of lymphoma) [20]. Dogs have undergone major selection pressure by humans for hundreds, if not thousands, of years, resulting in genetically distinct groups. Most cat breeds developed within the past century and there are fewer genetically distinct breeds than in dogs [21,22]. The fact that there are very few specific breed predispositions reported for cancer in cats may reflect this, although Siamese cats are over-represented in cats with cancer in general, and specifically with mammary gland and intestinal neoplasia [23,24,25]. The recently available feline genome may allow identification of genetic signatures of cancer risk in Siamese compared to other cats.

In terms of using cats as pre-clinical models for assessment of new drugs, thorough consideration needs to be given to drug metabolism and pharmacokinetics in this species before determination of applicability in people can be made. Cats are known to have reduced overall hepatic glucuronidation capacity compared to dogs and humans, though this seems to be drug-specific [26,27]. Consideration of metabolic pathways prior to clinical trials of potential new drugs in cats is recommended. Examples of chemotherapeutic drugs with differing toxicities in dogs and cats include cisplatin (fatal pulmonary edema in cats) [28], 5-fluorouracil (fatal neurotoxicity in cats, which is not due to reduced DPD function) [29], CCNU (lower apparent risk of hepatotoxicity in cats) [30], doxorubicin (nephrotoxic in cats, cardiotoxic in dogs) [31] and ifosfamide (higher maximum tolerated dose in cats) [31].

Despite the potential challenges, investigating cancer in cats is likely to add to the field of comparative oncology and complement the use of the canine model, especially in some specific instances.

## 2. Feline Oral Squamous Cell Carcinoma

Squamous cell carcinoma represents 70–80% of all oral tumors in domestic cats. Increased risk has been associated with exposure to environmental tobacco smoke, flea collars and feeding canned food [32], although specific risk factors are not identified in most patients. Tobacco use, along with alcohol, is a major risk factor in human head and neck cancer (HHNC), most of which are SCC affecting the oral/oropharyngeal cavity [33,34]. Papillomavirus infection plays a role in a significant proportion of HHNC, and is associated with less aggressive disease and better outcome [34]. Although papillomavirus may play a role in feline cutaneous SCC and has been isolated from oral papillomas in cats, it has not been demonstrated to be involved in the vast majority of feline oral SCC [35,36,37,38]. In humans, it seems that many oral/oropharyngeal SCC arise from pre-existing disorders such as leukoplakia and erythroplakia [34]. Such progression has not been identified in feline oral SCC, although inflammatory conditions like periodontal disease and stomatitis are common [39]. It is possible that there are similar pre-malignant lesions in cats which, if identified, may be models for chemoprevention or other intervention strategies. In both humans and cats, head and neck SCC is locally invasive, often diagnosed late, and is challenging to treat [33,34,40]. In cats, local disease is typically life-limiting and metastasis is less commonly reported than in humans, though may be underestimated since survival times are short because of advanced stage at diagnosis and poor response to local therapies [33,40]. The most common laboratory models for HHNC are induced tumors in the oral cavity of hamsters or rats, which have shed light on tumor initiation by carcinogens such as tobacco or betel nut, and subcutaneous xenograft models [41]. Feline oral SCC more closely mimics the natural behavior of HHNC and likely more accurately predicts response to treatment than induced tumors in laboratory animals.

As well as clinical features, feline oral SCC shares many molecular features with HHNC, including high frequency of epidermal growth factor (EGFR) over-expression (although not proven to be prognostic in cats) [34,42,43,44,45,46], altered p53 expression [34,47,48], dysregulated CK2 expression [49,50], markers of angiogenesis [34,46,51,52] and cyclooxygenase and lipoxygenase enzyme overexpression [34,53,54,55,56].

Feline oral SCC (either in clinical patients, cell lines or immunohistochemical studies) has been used as a model for tumor hypoxia (which may be targeted to improve responses to chemo- or radio-therapy) [57] and mechanisms and treatment of bone invasion [51,58,59,60,61]. EGFR is a druggable target in HHNC [42], and the feline and human EGFR sequences are highly homologous [10]. The EGFR small molecule inhibitor gefitinib is approved for use in HHNC, but has only a modest effect as monotherapy and, even in patients that initially respond, resistance commonly develops after long-term treatment [62]. Gefitinib resistance has been evaluated in a feline SCC cell line [10,63] and the cat could be a useful model for studying mechanisms of, and strategies to circumvent, EGFR-inhibitor resistance (e.g., RNA interference) [10]. Increased polyamine content is a feature of many tumors in humans, including HNC, and targeting polyamine synthesis with ornithine decarboxylase (ODC) inhibitors has been proposed as a possible therapeutic strategy [64]. A combination of alpha difluoromethylornithine (DFMO), an ODC inhibitor, with a novel membrane transport inhibitor to increase intracellular DFMO concentrations showed positive results in a murine model, with a 71% complete response rate [65]. In feline patients with naturally occurring oral SCC there was some indication of activity, but results were more modest (16.7% partial response rate, 50% stable disease). This may offer more realistic expectation of what might be expected from this type of therapy in people [66]. Inhibition of CK2 is effective in rodent xenograft models of human prostate and head and neck cancers [67,68,69] and small molecule CK2 inhibitors are in early clinical trials. *In vitro* results in feline SCC show similar results to human cell lines [50], and a clinical trial of RNA interference targeting CK2 is underway in cats with oral SCC, which may inform future human studies.

Overall, current standard treatments for feline oral SCC (surgery, radiation and chemotherapy) have almost universally poor outcomes, with median survival times in the order of a few months [40,70,71]. Better results are seen in small tumors [71], but as most patients have advanced disease at the time of diagnosis, treatment is typically palliative. With the grave prognosis and lack of effective standard of care, it is reasonable to offer experimental therapies to cats and owners at the time of diagnosis, and therapies can be assessed in a treatment-naïve population who may be more likely to respond. Potential avenues for comparative investigations in feline SCC include new EGFR inhibitors, including in the setting of gefitinib-resistance, CK2 inhibition alone and in combination with chemo- or radio-therapy, novel COX/LOX inhibitors, methods to reverse hypoxia in combination with other therapies, and anti-angiogenic therapies. Toceranib phosphate (Palladia, Zoetis) is a multi-kinase inhibitor which has shown some anecdotal efficacy in feline oral SCC. It does not inhibit EGFR, so the mechanism of its activity is currently unknown, and an investigation currently underway evaluating expression of toceranib targets in feline oral SCC [72] may identify new targets in HHNC as well.

## 3. Feline Mammary Gland Tumors

In cats, unlike dogs, the vast majority of mammary gland tumors are malignant, and multiple tumors and metastasis are common at diagnosis [73,74,75,76]. Thus, cats with mammary cancer may offer a larger population of aggressive malignancies to study. The epidemiology of mammary gland tumors in cats and people is similar, with age [23,74] and hormone exposure [73,77] being major risk factors. There is a breed predisposition in Siamese cats, which are more likely to develop mammary tumors and at a younger age than other cat breeds [23,73]. Now that the feline genome is available, Siamese cats may be a model for genetic risk of breast cancer and other neoplasia, given their increased risk of several tumor types. Germline mutations in BRCA1 and BRCA2 genes are associated with familial breast cancer risk in women, although the majority of breast cancers are sporadic in nature [78]. BRCA mutations have not been found in cats with mammary cancer [79]. Since there is a breed predisposition (if not a proven inherited risk), studying Siamese cats specifically may be more likely to identify these or other genetic abnormalities predisposing to mammary cancer.

In contrast to breast cancer in women, feline mammary tumors are more likely to be hormone (estrogen and progesterone) receptor negative, though differing methodologies and scoring makes comparisons between studies challenging [80,81,82,83,84]. Epidermal growth factor receptor 2 (HER2, neu, erbb2) is commonly over-expressed in human breast cancer and is a druggable target, with trastuzumab (Herceptin^®^, Genentech) improving outcome in women with HER2-expressing breast cancer [85]. Increased HER2 expression and activity, demonstrated by increased downstream AKT activation, is also seen in feline mammary carcinomas, though there is variation among studies with regard to the rate of HER2 expression and methodologies used [79,84,86,87,88,89,90]. Recent studies have used the human standard methodology (HercepTest^TM^, Dako) for evaluation of HER2 in feline mammary tumors [87,90,91] which may offer a useful standard for future studies. Concurrent evaluation of HER2 mRNA expression (as well as protein expression) may add to the understanding of its role in feline mammary gland tumors, though currently published studies are discrepant in terms of relative HER2 expression between normal and neoplastic tissues [11,84,91]. There appears to be a significant proportion of feline mammary carcinomas which are ‘triple-negative’ *i.e.*, hormone receptor negative and not over-expressing HER2 [79,84]. This phenotype is generally associated with a poorer prognosis in humans and is challenging to treat because of a lack of specific targets. Additional molecular analyses distinguish several other subtypes of breast cancer, e.g., luminal A, luminal B and claudin low, and similar subtypes may exist in cats [84,92]. The overall increased likelihood of aggressive mammary cancer in cats compared to humans offers opportunities to study factors such as drivers of metastasis and potential treatments for triple-negative tumors. Cats may also be a useful model for evaluating new HER2-targeted therapies. A recent study vaccinated healthy cats with HER2 DNA and induced specific T cell responses to self-HER2 in four out of 10 cats [93]. Cats with naturally occurring mammary cancer could be used for rapid pre-clinical assessment of efficacy of HER2 vaccination as well as to assess determinants of response, given the variability in induced immunity. When considering cats as a model for HER2-targeted therapies however, strong consideration needs to be given to the methodologies used to assess HER2 protein expression due to variability between studies, as well as the recently identified sequence variants in HER2 which may impact its affinity for targeted therapies such as trastuzumab, as is seen in humans [91].

## 4. Injection Site Sarcoma

Inflammation is well-established as a risk factor for several cancers in people and similar risk factors likely exist in dogs and cats [94,95]. Although there are sporadic cases of sarcomas associated with various implants and injections in humans and animals [96,97,98,99,100], cats appear to have a unique propensity for sarcomagenesis associated with trauma and/or inflammation. Injection site sarcoma (ISS) is a well-recognized phenomenon in cats, especially in association with vaccine administration. Estimates in the US and UK range from one case of ISS per 1,000–12,500 cats vaccinated [101,102]. Most ISS in cats are fibrosarcomas, and common histologic features include multinucleate giant cells, myofibroblastic differentiation and inflammatory infiltrate (lymphocytes and macrophages), which are not typically seen in feline non-injection site fibrosarcomas [103,104,105,106]. Intra-ocular sarcoma formation following trauma or other ocular disease is also recognized in cats [107]. No risk factors have been identified to determine why some cats develop sarcomas following trauma or injection. It is hypothesized that in these cats the immune response to injection or trauma is inappropriate and excessive, resulting in chronic inflammation causing proliferation and malignant transformation of fibroblasts. Chronic inflammation increases the risk for many carcinomas in people, though the only clear association between sarcomagenesis and chronic inflammation is in the case of Kaposi’s sarcoma (KS). KS is frequently associated with inflammatory infiltrate and Kaposi sarcoma-associated herpesvirus (KSHV) proteins activate factors including Th2 lymphocytes, cyclooxygenase 2 and NFκB resulting in a pro-tumor inflammatory microenvironment [108]. ISS in cats may model inflammation-associated tumorigenesis in general, which likely has common pathways in many different tumor histologies. As not every cat vaccinated develops ISS, not every person with chronic inflammatory disease develops associated cancer. Given that lifestyle factors such as environment and diet are likely more controllable in cats than in people, genomic screening in cats that develop ISS may identify features of the immune system or other factors related to tumor development. Such features may also be present in humans, and could identify individuals who may benefit from early intervention or more frequent monitoring. As well as a model for identifying risk factors, ISS may be a model for prevention or therapy. A recent development in ISS treatment is the approval of feline interleukin-2 recombinant canarypox virus (Oncept Il-2) to reduce local recurrence following standard-of-care therapy [109], indicating that manipulation of the immune system in this, and other inflammation-driven tumors, may be of therapeutic benefit. Further elucidation of the nature of feline ISS-associated inflammation (e.g., assessing cytokine profiles, T cell subtypes, and tumor-infiltrating macrophages) may be a first step in identifying strategies to reduce tumor-promoting inflammation and/or promote anti-tumor inflammation. Potential chemoprevention strategies could likely be evaluated in a relatively timely fashion since it appears that most ISS develop within 3 years of vaccination, though latent periods of up to 10 years are reported [110,111].

## 5. Conclusions

Although a decade behind their canine counterparts, cats have great potential to contribute to comparative oncology. Areas of especial focus may include head and neck squamous cell carcinoma, aggressive mammary tumors and inflammation-associated tumorigenesis. Areas for progress in order to exploit the full potential of the feline model include standardization of target assessments, development of efficient genome-wide analyses (e.g., feline-specific microarrays) and education of the public and veterinary communities about clinical trials and other comparative opportunities.

**Table 1:** Feline squamous cell carcinoma, mammary gland carcinoma and injection-site sarcoma cell lines used in published research.

Tumor Type	Cell Line
Head and neck squamous cell carcinoma	SCCF1 [112] SCCF1-Luc (luciferase-expressing) [47] SCCF1G (gefitinib-resistant) [10]
	SCCF2 [58] SCCF2-Luc
	SCCF3 [58] SCCF3-Luc
Mammary gland tumor	K12 [113]
	JM [114]
	FYMp (primary) [115]
	FKNp [115]
	FNNm (metastatic) [115]
	FONp [116] FONm [116]
	FMCp1 [115] FMCp2 [115] FMCm [115]
	FRM [117]
	NAC [118]
	K248C [119]
	K248P [119]
	DT09/06 [120]
Injection site sarcoma	FSA [121]
	FSB [121]
	FS1 [122]
	FS2 [122]
	FS3 [122]
	FS4 [122]
	VAS-1 [123]
	VAS-2 [123]
	VAS-3 [123]
	VAS-4 [123]
	VAS-5 [123]
	JB [124]
	JBLM [124]
